# The Projective Consciousness Model and Phenomenal Selfhood

**DOI:** 10.3389/fpsyg.2018.02571

**Published:** 2018-12-17

**Authors:** Kenneth Williford, Daniel Bennequin, Karl Friston, David Rudrauf

**Affiliations:** ^1^Department of Philosophy and Humanities, University of Texas at Arlington, Arlington, TX, United States; ^2^Department of Mathematics, Mathematics Institute of Jussieu–Paris Rive Gauche, University of Paris 7, Paris, France; ^3^Wellcome Trust Centre for Neuroimaging, University College London, London, United Kingdom; ^4^Faculty of Psychology and Education Sciences, Section of Psychology, Swiss Center for Affective Sciences, Centre Universitaire d’Informatique, University of Geneva, Geneva, Switzerland

**Keywords:** consciousness, first-person perspective, projective geometry, active inference, Free Energy principle, perspectival imagination, neurophenomenology, cybernetics

## Abstract

We summarize our recently introduced Projective Consciousness Model (PCM) (Rudrauf et al., 2017) and relate it to outstanding conceptual issues in the theory of consciousness. The PCM combines a projective geometrical model of the perspectival phenomenological structure of the field of consciousness with a variational Free Energy minimization model of active inference, yielding an account of the cybernetic function of consciousness, viz., the modulation of the field’s cognitive and affective dynamics for the effective control of embodied agents. The geometrical and active inference components are linked via the concept of projective transformation, which is crucial to understanding how conscious organisms integrate perception, emotion, memory, reasoning, and perspectival imagination in order to control behavior, enhance resilience, and optimize preference satisfaction. The PCM makes substantive empirical predictions and fits well into a (neuro)computationalist framework. It also helps us to account for aspects of subjective character that are sometimes ignored or conflated: pre-reflective self-consciousness, the first-person point of view, the sense of minenness or ownership, and social self-consciousness. We argue that the PCM, though still in development, offers us the most complete theory to date of what Thomas Metzinger has called “phenomenal selfhood.”

## Introduction

Everyone experiences the world from a situated, first-person point of view. We perceptually sample the world synchronically and diachronically via multiple, spatially distributed sensory organs, but the multifarious conscious perceptions typically related to the stimulation of these different sensory organs are all integrated into one coherent experience of one’s ordered spatial environs, with oneself at the center. And this spatial organization is not simply a matter of perception: imagination, anticipation, reasoning, action planning and execution, attention modulation, affective appraisal, and social cognition are all normally experienced as working in seamless concert with the perspectival space of conscious perception. Can we describe this integrated, multimodal and “centered” spatial structure of conscious experience in a rigorous way? And can we give a good account of why it should be thus organized? Here we answer both questions in the affirmative.

We maintain that the perspectival organization of consciousness is a highly non-trivial feature. It is essential to both the general phenomenological structure of consciousness and the cybernetic (or “control theoretic”) functional role consciousness plays for the embodied organism. This dual—phenomenological and functional—conception of the perspectival structure of consciousness is the starting point of our recently introduced Projective Consciousness Model (PCM) (Rudrauf et al., 2017). The PCM postulates that the mind is a process mediating active inference supporting navigation in and learning from the environment in a globally optimal manner (Friston et al., 2015, 2016; Friston K. et al., 2017). The PCM combines a model of cognitive and affective dynamics based on variational and expected Free Energy (FE) minimization with a model of perspective taking [or a “Field of Consciousness” (FoC) embedding a point of view] based on 3D projective geometry.

We argue that projective geometry accounts for certain puzzling features of consciousness that stem from its perspectival character (e.g., the elusiveness of the point of view, cf., Howell, 2010; Williford, 2010; Williford et al., 2012; Renaudie, 2013) and is essential to the full explanation of its key cognitive-behavioral functions. The concept of 4D projective transformation is central to the model, as it yields an account of the link between perception, imagination, and multi-point-of-view action planning. Overall, the PCM delivers an account of the phenomenologically available, generic structure of consciousness and shows how consciousness allows organisms to integrate multimodal sensory information, memory, and emotion in order to control behavior, enhance resilience, optimize preference satisfaction, and minimize predictive error in an efficient manner.

We briefly present our methodology (see the section “Methodology”), present the PCM (see the section “Phenomenological Invariants and Functional Features”) and then discuss some outstanding philosophical issues in the light of the model (see the section “The ‘Hard Problem’, Representationalism, and Phenomenal Selfhood”). In this latter section, we argue that, though metaphysically neutral, the PCM fits well into a (neuro)computationalist framework, that it does not fallaciously infer consciousness’ spatial structure from the conscious representation of space, and that it helps to distinguish as well as integrate sometimes conflated or ignored aspects of subjective character, including pre-reflective self-consciousness, the first-person point of view, the sense of minenness or ownership, social self-consciousness, and empirical and “transcendental” egological structures. We suggest that the PCM offers the most complete theory to date of what Thomas Metzinger has called “phenomenal selfhood” (Metzinger, 2003; Blanke and Metzinger, 2009).^1^

## Methodology

We approach consciousness as a natural phenomenon that requires a well motivated, parsimonious, generative (mathematical) model of observable conscious phenomena, based on general principles and postulates and yielding empirically testable predictions.

Modeling consciousness first demands a “phenomenology” of consciousness, a rigorous description of its reflectively available invariant^2^ structures. We take this to be part of the *neuro*phenomenology program (see Varela, 1996; Rudrauf et al., 2003), which contains the following mutually constraining components:

(1) Observation and description of the phenomenological invariants at the appropriate level of abstraction.
(2) Classification of the behavioral, cognitive, and biofunctional aspects of consciousness.
(3) Identification of the neuroanatomical and neurophysiological correlates of consciousness.
(4) Development of mathematical and computational models of consciousness that (a) faithfully integrate all the invariants described in (1), (b) provide unifying explanations of and testable hypotheses about the biofunctional aspects of consciousness described in (2), and (c) can be physically implemented in the “hardware” identified in (3), as well as perhaps in non-biological hardware.

Ideally, this method would give us a theory of consciousness that intelligibly links the phenomenology of first-person conscious experience with its realization in the brain (or other substrate) via a mathematical and computational model and simultaneously accounts for its biofunctional properties.

The PCM starts from an attempt to formalize phenomenological features characteristic of consciousness by describing them in terms of general mathematical principles and frameworks (viz., projective geometry and FE minimization). Such principles and frameworks then provide us with strong constraints for further developing the model in order to account for specific phenomena (e.g., specific visual illusions, approach-avoidance behaviors related to imaginary perspective taking). In other words, the PCM must be seen, above all, as a model or theory of the *generic*, invariant structures and functions of consciousness. Specific experiential or experimental conditions, combined with the general model, yield specific predictions. Much work remains regarding the application of the general model to the variety of such specific conditions for empirical testing. We have worked out several cases, and are in the process of testing them further (see examples referred to below).

## Phenomenological Invariants and Functional Features

### Phenomenological Invariants

The following are the deeply interconnected invariants that we think characterize consciousness.^3^ We take them as postulates for mathematical treatment (cf., Oizumi et al., 2014) and construct the PCM on their basis:

(1) *Relational Phenomenal Intentionality*: All consciousness involves the appearance of a world (of objects, properties, etc.), in various qualitative or representational ways, to an organism.
(2) *Situated 3D Spatiality*: The space of the presented world (objects, etc.) is 3-dimensional and perspectival, unfolding in an oriented manner between a point of view and a horizon at infinity (where all parallel lines converge). The origin of the point of view is elusive, though it normally seems to be located in the head. The space is normally organized around the lived body.
(3) *Multimodal Synchronic Integration*: Consciousness involves the synthesis or integration into a unified whole of a multiplicity of qualitative and representational components (from sensory modalities, memory, and cognition).
(4) *Temporal Integration*: Consciousness involves at least the retention of immediately past experiences and the protention of immediately future experiences, as integral elements of its “specious present” and a foundation for more expansive forms of temporal integration (distant memories, long-term plans, etc.).
(5) *Subjective Character*: Consciousness involves a pre-reflective, non-conceptual awareness of itself and its individuality.

We hold that (2)–(5) are, in fact, all implicated in (1).

*Intentionality* (1) encompasses four conceptually distinct but intertwined features: (i) representational content, (ii) qualitative character, (iii) perspectival character, and (iv) subjective character. Consciousness seems to involve the perspectival presentation of something (an object, quality, or state of affairs) *to* the conscious system in some specific way and in a situated context (or “world”). We maintain that *all* consciousness is at root relational in its structure in a manner that is at least proto-spatial and is always framed around a point of view. This includes the experience of all types of sensory qualities as well as basic affective and mood states and even cognitive phenomenology.^4^

*Spatiality* (2) refers, more specifically, to the fact that the space of experience is 3-dimensional and perceived (or imagined) from a situated, first-person point of view centered on the lived body by default. *Spatiality* (2) is perhaps most easily noticeable in visual experience, though careful attention will reveal that it integrates the vestibular and proprioceptive senses, which are in fact necessary for the vision-centered localization of the point of view, as well as all other sensory modalities.

The point of view or origin is elusive, phenomenologically speaking. It is usually roughly localizable as being behind the eyes; but in out-of-body experiences (OBEs) and other types of abnormal consciousness, it can seem to be elsewhere (Lenggenhager et al., 2007; Blanke and Metzinger, 2009) or strikingly ambiguously located [as in heautoscopy (Sacks, 2012; Blanke et al., 2016) and some manifestations of schizophrenia (Fuchs, 2018)]. It is elusive because it does not appear to the experiencer as a distinguishable object or precisely localizable point within the phenomenal space.^5^ Rather, it functions as an innermost zone around which experience is organized. We see objects in the distance *from here*, but we could see them *from over there*. If we change our location to be able to see something closer up or from a different vantage point, the elusive origin moves with us. The origin of the point of view in our 3D experiential space is elusive and implicit in the way the origin of 2D planar perspective drawings and photos is: the point of view is not itself an object in the photo or drawing, but it is indeed a condition for the generation and comprehension of the picture (cf., Van Gulick, 2004; Velmans, 2009, 140ff.).

*Multimodal Synchronic* (3) and *Temporal Integration* (4) are similar in that they both involve the fusion or integration of differences into a whole, unified, coherent episode and, respectively, sequence of episodes of consciousness. One sees, hears, smells, feels, thinks about, etc., many qualities and objects simultaneously as well as diachronically. Among other ways, this integration manifests spatially by the fact that there is normally no sense of discontinuity between the boundaries of the visual field and its multimodal experiential complement all around. In the temporal domain, one’s current episode of consciousness is colored by what one has just experienced (retention) and what one implicitly anticipates (protention) in the immediate future (see Husserl, 2012). Temporal integration can involve longer and shorter spans, and we do not pronounce upon what exactly the minimal span is. We will not treat *Multimodal* and *Temporal Integration* in detail in this article, but we claim that both diachronic and synchronic integration of information are framed in consciousness by a perspectivally structured space [and thus relate essentially to *Spatiality* (2)].

*Subjective Character* (5) will be discussed in some detail in the section “The ‘Hard Problem’, Representationalism, and Phenomenal Selfhood”, but it should be clear that it relates to (1) since relationality suggests a subject relatum, to (2) since the point of view is experienced to be that of the individual conscious subject, and to (3)–(4) in that synchronic and diachronic integration seem to center around this same point of view or a sequence of points of view connected by retention and memory.

### The Invariants and Projective Geometry in the PCM

Here we want to motivate our appeal to projective geometry. The concrete embodiment of consciousness entails location and limitation or situatedness, which is a key property in the derivation of the PCM (Rudrauf et al., 2017). Our bodies can only occupy one portion of space at a time, and we can never see an object from all points of view at once. We routinely integrate perceptions mediated by spatially distributed sense organs, but we nevertheless have to deal with the surrounding world by adopting a series of perspectives on it.

Perspectival spatiality is thus, in our view, a general or determinable feature of every determinate, particular, situated conscious experience.^6^ For instance, to experience one’s limbs as occupying a certain location in space in relation to other objects in the environment and the rest of one’s body, one must, in the first place, experience a whole phenomenal *space* which subsumes the experienced location of those limbs and their other possible spatial positions. Moreover, that space must be centered around an origin in order to define the set of possible locations, distances, directions, and spatial relations of potential interest to the situated agent. This perspectival or egocentric frame (cf., Campbell, 1994) defines the spatial component of what we call the FoC. The traditional terminology of a “FoC” (cf., Gurwitsch, 1964) or “phenomenal field” (cf., Bayne, 2010) is quite apt here in two senses: there is the spatial character of experience, which is organized like a 3D egocentric vector field (we have a sense of direction and motion all around us); and consciousness behaves somewhat like a force field attracting attention and driving action toward objects and locations in 3D, under the influence of an affective dynamics (see below).

If the FoC is perspectival (has a point of view), it cannot be Euclidean in its geometrical structure. It therefore must be conceived of as being different from the surrounding physical space from the outset. This strongly suggests that it must also be *virtual*, in roughly the sense in which the word is used in “Virtual Reality” (cf., Velmans, 2009, 140ff.; Rudrauf, 2014). Our intended meaning is perhaps most clearly manifest in phantom-limb phenomena: one intuitively experiences the phantom limb as being in a certain location in space; but there is no limb in the physical location the phenomenal limb is mapped to; hence the phenomenal bodily space is *not* the ambient physical space, even though there is a functional mapping of the former onto the latter that must be accurate enough most of the time for practical purposes.

This perspectival space of the FoC is organized around an elusive, implicit origin or “zero-point” (cf., Merker, 2013; Horgan and Nichols, 2016; Kapitan, 2016) and includes a horizon with vanishing points “at infinity” that mark its limit. The FoC embeds “vectorized” directions that frame the orientation of attention and action along three axes (vertical, horizontal and sagittal). These are given in perspective with respect to these same points at infinity. This is most evident in the case of vision (e.g., think of converging railroad tracks), but it applies, we believe, to conscious auditory and tactile spaces (at the very least, they are normally integrated with the space of vision). It also applies to imagination, as when we take imagined perspectives on objects from points of view we do not currently occupy. In the FoC, objects appear (e.g., visually or audibly) to “grow or shrink” as they approach or recede, while their size is usually taken to be constant. Similarly, stable objects are normally taken to have constant shapes (e.g., a rectangular table) but have variable apparent shapes (e.g., trapezoidal) depending on the angle of view.

This perspectival phenomenal space allows us to locate ourselves and navigate in an ambient space that is nevertheless essentially Euclidean. However, in Euclidean space, there are no privileged points, no points at infinity, no horizon, and no non-arbitrary origin. This very strongly suggests that a non-Euclidean frame must be operating, with the help of sensorimotor calibrations, to preserve the sense of the invariance of objects across the multiple perspectives adopted and relate the situated organism to the ambient Euclidean space.

Since we are dealing with a 3D perspectival space, with an (elusive) origin and vanishing points at infinity constituting a horizon plane, the PCM postulates that the FoC has the structure of a 3D projective space in the sense of Projective Geometry^7^. The FoC can thus be partly understood in terms of a 3D *projective frame*.^8^

A projective space requires an origin of projection outside of that space. This is obvious for a projective 2-space as implicated in drawings “in perspective”, which involve a projection onto a plane through an origin of projection outside the plane along a third dimension. It is a fundamental definition of projective geometry that a projective space of *n* dimensions implies an origin of projection outside the *n*-space, residing within an *n+1* vector space. A related theorem is that any projective transformation in *n* dimensions can be interpreted as a composition of perspectives in a projective space of *n+1* dimensions. A projective 3-space thus necessarily involves a projection onto a 3-space through an origin outside of the space along a fourth dimension.

The origin of projection cannot appear in the FoC as an object in the space. Rather, it can only appear elusively, encoded in or implied by the geometrical structure of the FoC. Nevertheless, the existence of a fourth dimension is a necessary consequence of the projective setup. However, it must not be understood here as an additional physico-spatial dimension but instead as a parametric dimension in an abstractly describable vector space. This is unproblematic from a computationalist point of view. In fact, the necessity of invoking a fourth vector dimension supports a computationalist account of consciousness (see the section “The ‘Hard Problem’ and Computational Functionalism” below).

A further implication of this model is that all transformations of points of view, all perspective taking (whether in perception, imagination, or action programming) must be governed by changes of frames described by the Projective Linear Group in four dimensions acting on lines in a 4D vector space, which, for practical purposes, can be expressed as 4 × 4 transformation matrices applied to homogeneous coordinates.

We thus must conclude that a particular extension of three-dimensional space (viz., 3D projective space), which is independent of (but may incorporate) metrical notions, is required for generating consciousness. It extends the mostly unconscious Euclidean geometry used for motion preparation and execution and underlies the more consciously accessible geometry used for navigation. 3D projective geometry is the geometry of points, lines and planes that takes into account only natural incidence properties. It is the geometry of sheaves of lines or oriented lines issued from points, and this forms the ground for experiencing directed actions and motions and for taking multiple points of view. According to the PCM, this shift to a projective geometry is necessary for the consciousness.

We will discuss further how the geometrical component of the PCM relates to other puzzles of phenomenal selfhood and subjectivity in the final section. Here we emphasize its connection to perspectival imagination and intersubjectivity (see also Rudrauf et al., 2017).

Perspectival imagination—being able to know or infer, for example, how things would look from a different vantage point—is central to our very “being-in-the-world.” Here, we want to emphasize that there is a kind of tacit, “working” perspectival imagination that configures normal perceptual experience and reflects a projective geometry. The Husserlian description of “emptily intending” the unseen parts of physical objects illustrates the point (see, e.g., Husserl, 1982, 2012). Empty intending, which is fused with normal perception, is the basis of our normal anticipations (“protentions”) with respect to familiar objects. I can, for example, focus my attention on the location just behind a door, expecting to see a person as I open it; I can turn a book over to verify what is on its back cover, tacitly expecting to find a backside with something on it (words, colors, pictures). We could not aim beyond the immediately visible and tangible aspects of the door and could not understand the book as an 3D object, if we did not have the capacity to (tacitly) imagine other accessible but currently non-actual points of view (see Husserl, 2001; Madary, 2016; Williford, 2017). This sort of working perspectival imagination is constantly operative in normal perceptual experience and is framed by projective transformations.^9^

We can also more explicitly and deliberately imagine what new vantage points might reveal and routinely do so. We can pass from a current perspective on an object to “what it would look like from over there” or what ordinary objects of familiar shape and size ought to look like as we approach them, recede from them, or as they (or we) rotate (*modulo* certain empirical constraints, see Pizlo, 2008). Importantly, the imagination of objects in space outside the visual field also respects projective rules (e.g., imagine an object behind you and move it away, it will appear smaller as it is farther away and vanish at infinity). Moreover, the group of projective transformations [PGL(4), for projective 3-space] contains the group of displacements of rigid objects, and thus a permanent shift or superposition of transformations enables the representation of voluntary movements of the body in addition to the motions of objects.^10^ This ability for perspective taking, which echoes voluntary movements in the world (cf., Berthoz, 2000), is inexplicable without projective geometry; and the affordances offered in any metric space would be impossible to represent without a projective geometry.

Importantly, because we routinely truck in actual and possible vantage points, we come equipped to conceive of vantage points other than but possibly related to our own (cf., Husserl, 1991, p. 53). Thus perspectival imagination endows us with one of the bases for intersubjectivity, which is of obvious biological import (see, e.g., Humphrey, 2002; Cronin, 2005). Perspectival imagination and intersubjectivity thus appear grounded by the group of projective transformations, which fact, in turn, reinforces our confidence that 3D projective geometry is essential for modeling the structure of consciousness.

**FIGURE 1:**
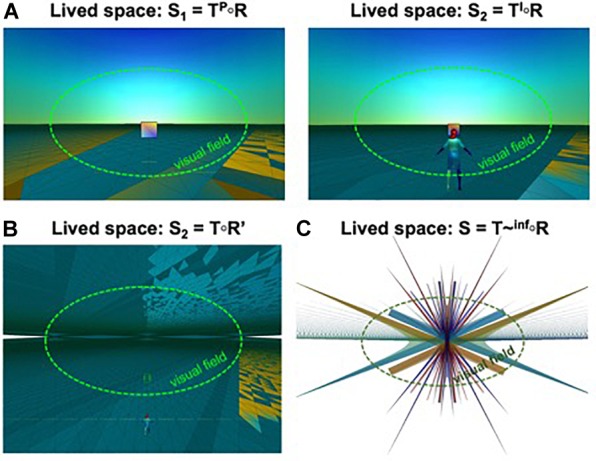
Account of psychological phenomena. **(A)** Projective solution to VR-mediated dissociations in phenomenal selfhood. The visual field is indicated (dashed-green line) as a subset of lived space. **Left**: Projection from the normal perception spectrum: *S*1 = *T P* ∘ *R*, the projective frame is calibrated in first-person perception-mode. **Right**: Third-person projection from the pure imagination subgroup: *S*2 = *T I* ∘ *R*. Perceptual experiences of dissociation between the subjective point of view and the sense of location of the body induced by VR experiments using sensory conflict are explained by the PCM as the result of a projective solution that is not normally used for perception but is chosen as the best explanation of sensory data. **(B)** The anti-space beyond the plane at infinity. Rendering of the projective space with a transparent plane at infinity and no clipping, revealing the involution of the space mirroring the back of the ambient space intrinsic to projective spaces. **(C)** The “God’s eye” vantage point on the projective space. Rendering on a 2-dimensional image-plane of the projective space and its mapping of the world model R from the “God’s eye” point of view at infinity. Almost all the space is visible but completely warped and the world model manifests as structures with complex symmetries (from Rudrauf et al., 2017, used by permission from Elsevier).

**FIGURE 2:**
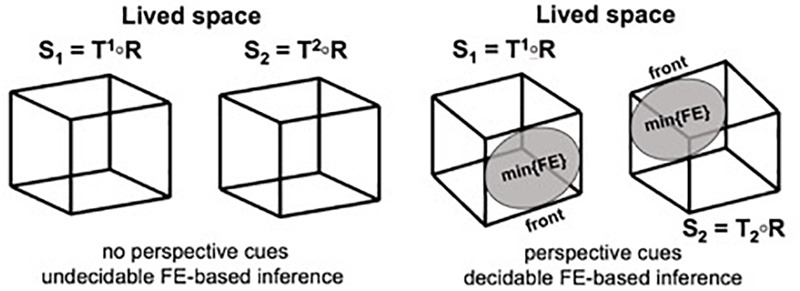
Projective solutions to the Necker cube. Projection of two complementary views *S*1 and *S*2 of a generic wireframe cube *R* in a projective space (rendered on an image plane). **(Left)** When the implicit point of view is placed far away and the projective frame has a narrow scope, no perspective information remains to disambiguate the orientation of the cube. Free Energy (FE) does not possess a unique minimum, each option is unsatisfying, the inference is globally undecidable but irrepressible, and the system can oscillate between the two possible outcomes. **(Right)** When the implicit point of view is located near the projected cube in ambient space, perspective information helps to disambiguate the inference about the two possible orientations of the cube, *T*1 and *T*2 (see gray ellipses indicating the front). As a result, FE always possesses a minimum for a unique transformation *T* and perception can stabilize (from Rudrauf et al., 2017, used by permission from Elsevier).

To summarize: according to the PCM, the geometrical structure of multimodal, lived conscious experience closely approximates a projective 3-space (e.g., RP^3^) on which projective transformations [e.g., PGL(4)] act. Our model fits intuitive data, but it also helps account for other phenomena that might seem at first unconnected, including working perspectival imagination, inferences about the points of view and aims of others (*modulo* active inference, to be discussed presently), and many other peculiar phenomena associated with consciousness (e.g., OBEs, heautoscopy, derealization, “oceanic” mystical experiences, see Rudrauf et al., 2017 and Figure 1). It also sheds light on previous accounts of well known visual phenomena, for instance, the multistable phenomenology of the Necker Cube (see Rudrauf et al., 2017 and Figure 2), and suggests new accounts of others, such as the Moon Illusion (cf., Rudrauf et al., 2018-preprint), that will be the topics of future communications.^11^

According to the PCM, to fully understand the phenomenology and functions of consciousness, one must also consider how projective geometry is the support of a general process of active inference (Friston et al., 2016; Rudrauf et al., 2017; Wiese and Metzinger, 2017), which entails approximate Bayesian inference under the FE principle (Friston, 2010). This is the second, central component of the PCM that we shall now discuss in relation to the functional or “cybernetic” role of consciousness.

### Functional Features of Consciousness

We claim that the overall function of consciousness is to address a general “cybernetic” problem or problem of control. Consciousness enables a situated organism with multiple sensory channels to navigate its environment and satisfy its biological (and derived) needs efficiently. This entails minimizing predictively erroneous representations of the world and maximizing preference satisfaction. A good model of consciousness must be able to explain how this is accomplished. The PCM thus emphasizes the following interrelated *functional* features of consciousness:^12^

(1) *Global Optimization and Resilience*: Consciousness helps the organism reliably find the globally optimal solutions available to it given its competing preferences, knowledge base, capacities, and context, making the organism more resilient. This is a mechanism of global “subjective” (here in roughly the sense of “subjective Bayesian”) optimization, which does not always entail “objective” optimality, though it must approximate the latter reliably enough (cf., Lewis, 1980).
(2) *Global Availability*: The contents of consciousness must be generally globally available (Baars, 1993, 1997; Dehaene, 2014) in the sense of “poised” to be treated of (cf., Tye, 1995) by our affective, cognitive, and perceptual systems as the occasion requires.
(3) *Motivation of Action and Modulation of Attention*: Consciousness is generally implicated in the motivation of action based on affective dynamics and memory (see Rudrauf and Debbané, 2018); this drives the modulation of attention (though consciousness is not to be narrowly identified with attention, cf., e.g., Koch and Tsuchiya, 2007 and Kentridge, 2011).^13^
(4) *Simulation Enhancement*: Consciousness facilitates the use of simulation (in the broad sense of “imagination” not the narrow sense of “simulation theory”) in the service of solving cognitive, perceptual, and affective problems, and more generally in the anticipation and programming of actions and the response to their outcomes.

It should be clear that, here again, these items are interrelated. Items (2)–(4) are, in fact, all either necessary for or highly conducive to item (1). Information that is not globally available cannot be dealt with using the full range of our capacities. If a person is unable to keep attention trained on salient objects in the environment, he or she will, at best, have difficulties properly navigating it and becoming familiar with the objects —this relates *Global Availability* (2) to *Motivation and Attention* (3). If an agent cannot simulate alternatives, then a whole range of possible solutions to problems becomes inaccessible, and they can become locked into brute-force, infantile, escapist or last-resort strategies, with severe psychopathological consequences and symptoms (cf., e.g., Fuchs, 2018)—this relates *Simulation* (4) to *Motivation and Attention* (3).^14^

Having a consciousness that incorporates a virtual, transformable projective space is a highly efficient way for an organism to keep track of its own location and orient itself in a multiplicity of real spaces—social spaces and time included. Likewise, projective geometry is necessary for an organism to be able to frame 3D perspectival salience maps, which are essential to the modulation of attention, appraisal, motivation, and orientation (see Rudrauf and Debbané, 2018). Conversely, the advantages and flexibility afforded by a projective geometrical framing would remain dormant were it not coupled with an appraisal engine capable of motivating behaviors based on the FoC.

### The PCM and the Functional Features—Active Inference and FE Minimization

The PCM combines projective geometry with an algorithm based on the principles of active inference (which embeds perceptual inference) driven by FE minimization in order to account for fundamental features of perceptual intentionality, object identification, and action guidance, based on cognitive and affective priors (Rudrauf et al., 2017; Rudrauf and Debbané, 2018, see also Friston, 2010; Frith, 2013; Hohwy, 2013; Clark, 2015; Metzinger and Wiese, 2017; Wiese and Metzinger, 2017). We choose to use FE minimization as the functional metric for optimization, since it has a solid conceptual history, and offers powerful solutions to the general problem of active inference for embodied agents in an approximate Bayesian framework (Friston K. et al., 2017).

Active inference is a method of information processing and control by which an autonomous system or agent (i) anticipates the consequences of its actions by predicting how they will affect the system perceptually, (ii) programs its actions and acts accordingly (given preferences), and (iii) (Bayesian) updates its prior beliefs based on a comparison between its predictions and sensory evidence in order to minimize predictive error.

Free Energy is a quantity transposed into Bayesian learning theory from statistical physics. It yields a functional for controlling mechanisms of predictive coding, directly applicable to active inference. The quantity can be expressed in different ways and can encode prior beliefs and preferences related to appraisal and behavioral policies and to past, present, and future expected states following actual or anticipated actions (Rudrauf and Debbané, 2018). Generally speaking, it can be formulated as the sum of two terms: (i) the the expectation of the difference between the logarithm of the probability of the prediction under a generative model encoding prior beliefs and preferences and the logarithm of the probability of the sensory outcome, and (ii) the departure of posterior beliefs from prior beliefs expressible by the Kullback-Leibler Divergence (KLD) (see, e.g., Friston, 2010; Friston K. et al., 2017; Wiese and Metzinger, 2017).

The FE principle entails that agents attempt to minimize their overall FE in order to maximize the accuracy of their beliefs and the satisfaction of their preferences in a globally optimal (or “all things considered”) manner. Formally, FE is an upper bound on “surprise”, which is key to assessing the predictive value of internal generative models of the causes of sensations. Note that the KLD in FE as expressed above can be developed into two terms: an expectation and a negentropy. When formulated that way, the form of FE appears closer to the form it takes in physics and better manifests its nature.^15^

An essential addition of the PCM to this paradigm is the framing of variational FE through the FoC. In the PCM, FE is a function of the action of projective transformations on the FoC so that the states (probabilities) and the weights defining FE depend on the choice of a projective frame F, which relates to where and how we look or aim at our surroundings (see Rudrauf et al., 2018-preprint). This applies to perception, recall, and imagination, which, notably, is used in evaluating anticipated actions through expected FE. The minimization of FE thus becomes a function of the FoC, that is, *of consciousness*. Thus consciousness becomes an integral part of a global optimization process for cybernetic control. What the PCM adds to all the approaches in the predictive coding, active inference and Bayesian Brain paradigms (see, e.g., Wiese and Metzinger, 2017) is this crucial geometrical component, yielding a psycho-functional model that is independent of the specific brain implementation.^16^

Projective Consciousness Model agents optimize the precision of their knowledge about the causes of their sensations and the satisfaction of their preferences based on the FoC, which frames the distribution of FE attached to objects and affordances across space and time, factually or by anticipation. Cycles of perception, imagination, and action, as well as prior updates are used to minimize FE as projectively framed. The agents combine perspective taking based on projective transformations and attributions of prior beliefs and sensory evidence (to themselves or other agents as the case may be) within a FoC in order to appraise the optimality of possible actions given a set of preferences. Because FE bounds surprise, expected FE places an upper bound on uncertainty. This follows because, mathematically, surprise is self-information, and expected self-information is entropy (i.e., uncertainty). In other words, the actions we anticipate are those that will resolve the greatest uncertainty. Note, however, that PCM agents simultaneously try to maximize preference satisfaction along a variety of dimensions of appraisal through FE minimization. Thus highly certain negative outcomes can be associated with a higher level of FE than some uncertain outcomes with possible positive results (cf., Rudrauf and Debbané, 2018).

To illustrate, in the PCM, your immediate conscious experience that seems to you to be the visual presentation of, for instance, a puppy curled up by the fire is the *projective framing* of your brain’s pre-personal, non-conscious, neurocomputational “best guess” as to what is causally responsible for your current and just passed sensory data in a given context and relative to certain priors (of varying flexibility) that constrain and bias your perception (cf., Merker, 2012).

Moreover, the consciously accessible “result” of these neurocomputational processes carries with it (partly consciously accessible) perspectivally framed predictions (“working imagination”) about the sensory data (and corresponding object perceptions) you are most likely to continue having or would have were you to take certain actions, thus serving as a support for further active inference. In this way, one does not end up only with a “God’s eye point-of-view” Euclidean map of the objects in ambient space, but something more like a “user surface” (Metzinger, 2003) that encodes all the projectively articulated structures we are so familiar with (e.g., the puppy seen from here versus there, partially occluded by the table, or seeing one coming toward it).

Active vision thus becomes like a “palpation” of a 3D spatial “user surface” to “feel” the epistemic affordances that are quantified by expected FE. This anticipatory palpation with temporal depth requires a generative model that encompasses the consequences of actions and their affective values. This requires executive functions such as working memory, the modulation of attention, deployment of cognitive resources, and affective appraisal dynamics that altogether enable us to minimize FE over alternative sequences of actions as necessary for maximizing expected values (see Friston K.J. et al., 2017; Rudrauf et al., 2017). The framing of expected FE through projective transformations in imagination provides a method for envisioning “how things would look, feel, be [etc.] from another, hopefully better, location”, and thus guide action. It is important to emphasize that according to the PCM, FE minimization is always operating as the fundamental algorithm governing the FoC, and thus there is never an end to the process, as there is never in practice a solution that reduces FE to zero. Hegel might be happy to know that on the PCM consciousness is thus always somehow “unhappy” even when it reaches the best possible states available to it.

The PCM also embeds a distinction between the FoC and what we call the “world model” (R). The world model is a generally unconscious (or “preconscious”) but accessible model stored in memory and included in the agent’s prior beliefs and generative models. It contains models of objects and relationships in the ambient Euclidean space across time and is continually updated on the basis of the agent’s history of perspective taking through a process analogous to multi-view reconstruction, based on reverse active inference and (epipolar) projective geometry (see Rudrauf et al., 2017). The current FoC accesses and contributes to updating the world model dynamically in a situated, perspectival manner (see Rudrauf et al., 2017). For example, when we consider a building or any structure in space, we not only perceive it in perspective, but also have a sense of it being a Euclidean object existing in independence of any particular point of view on it. This is accounted for by the combination of active inference and projective geometry and their role in building a world model. Thus, according to the PCM, it is projective geometry that optimally enables both the perspectival accessing of Euclidean models of the world in memory for appraisal and the updating of such models through active sampling so that one can build a (relatively) perspective-independent (objective) knowledge base. Projective geometry helps functionally connect subjectivity and epistemic objectivity allowing us to see consciousness as a kind of mediator between the situated organism and the objective world it inhabits.

**FIGURE 3:**
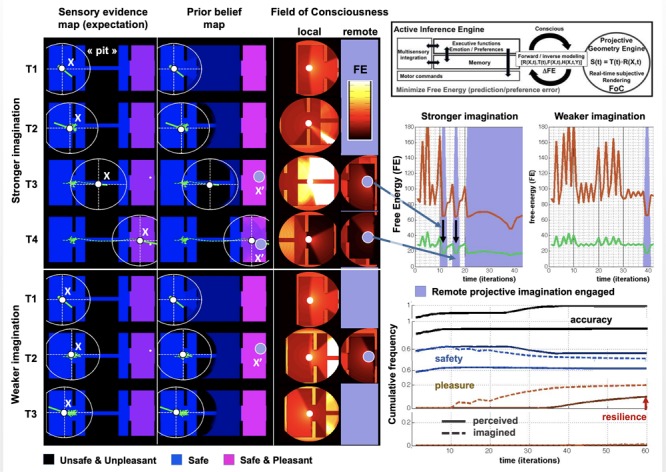
Simplified 2D PCM-based simulation of a pit challenge. **(Left Tier)** Maps of a 2D world model and agent with a departure room (left: safe and anhedonic) a challenge room with a pit (center: unsafe and unpleasant), a goal room (right: safe and pleasant) (see color code). Sensory evidence maps represent factual observations, prior beliefs maps, the beliefs of the agents, and large white circles their FoC. One simulated agent is more likely to imagine remote options (stronger imagination) than the other (weaker imagination). T1 to T4 stand for time periods. Circular FoC maps show ongoing perceived (local) and transiently imagined (remote) situations; colors index Free Energy (FE, see color bar). **(Right Tier, Top)** General PCM architecture: a projective geometry engine is controlled by a FE-driven active inference engine. **(Middle)** Time courses of FE. Optimal FoC perspectives have lowest FE (green line) compared to average of other possible perspectives (red line). **(Bottom)** Cumulative frequencies of agents’ appraisals across time and belief accuracy. A stronger imagination leads agents to cross the pit after hesitating and reach the goal room, maximizing utility, as goal-related, optimistic projective imagination reduces overall FE near the pit. As a result, there is a gain in pleasure or resilience versus initial conditions (red arrow), and exploration with prior updating increases accuracy. A lower imagination leads agents not to explore the environment, never enjoy a better condition or greater accuracy, and to repetitively wander locally. (See Rudrauf and Debbané, 2018 for more details on this particular family of simulations.)

**FIGURE 4:**
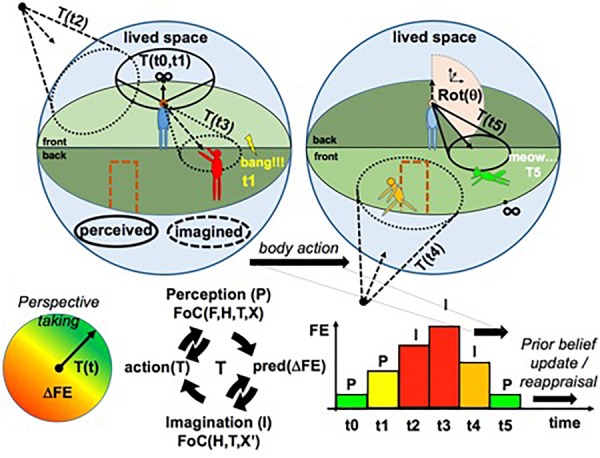
Overall sketch of the PCM. Lived space *S* (represented by blue spheres): subjective experience of space and its contents as perceived (continuous funnels on the figure) or imagined (dashed funnels), framed in a 3-dimensional projective representation in perspective of a world model *R*(*X, t*) associated with a distribution of Free Energy (FE), related to cognitive and affective personal preferences (colors on the manikins and door, and disk with color gradient). Perspective taking and the selection of corresponding projective transformations *T* are constrained by the gradient of FE across space δFE. The diagram with the arrows in a circle represents the possible transitions of state affecting the FoC: form perception to imagination and action. The FoC is partially driven by sensory evidence when it is focused on perception of the local environment and entirely driven by prior beliefs in memory and simulation capabilities when it is focused on imagination of non-local spaces (e.g., when we imagine ourselves at home while we are at work). FE minimization defines the optimal perspective at a given instant. FE is globally minimized through cycles of perception, imagination, and action across time. The bar graph represents FE as a function of time and of the different perspectives and processing modes [P—perception versus I—(non-local) imagination] adopted by the system (see text and Rudrauf et al., 2017; image from Rudrauf et al., 2017, used by permission from Elsevier).

To summarize: according to the PCM, each episode of consciousness is a computationally virtual and phenomenal space that approximates a 3D projective space (e.g., RP^3^). It is populated with representational content including affordances for the motivation of action at any given time in the manner described by the principles of active inference and FE minimization. These are the contents that are made “globally available” and thus are poised to be treated of by, in principle, all cognitive, affective, and perceptual modalities. Attentional modulation, affective responses and strategies, the deployment of cognitive resources, and the direction of behavior by working and deliberative projective imagination are all guided by the overall directive of global FE minimization (less technically, we generally prefer to be in or imagine pleasant and safe situations). The PCM thus hypothesizes that conscious brains should realize a projective geometrical rendering engine embedded in a general active inference engine, which in turn is presided over by a global FE minimization algorithm (Rudrauf et al., 2017) (see Figures 3, 4).

We note that unconscious activities have a broader scope that we cannot treat of here. Conscious and preconscious phenomena are indeed the tip of an iceberg, and we do not claim that the entire iceberg can be explained in these terms. Moreover, the inner geometry behind the 3D projective framing of the FoC is certainly much more complex, involving many variables that are difficult to describe precisely at present. What we say here applies primarily to consciousness, self-consciousness, and closely related preconscious dynamics.

Finally, we must emphasize that, as a scientific model, the PCM yields specific empirical predictions, in addition to offering a general explanatory framework that encompasses several well-known puzzling phenomena (see Figures 1, 2). The derivations of specific predictions from the general principles can be quite demanding mathematically (see, e.g., Rudrauf et al., 2018-preprint). Nevertheless, the PCM has been developed to make quantitative predictions about specific perceptual, affective, and behavioral phenomena. It makes quantitative predictions about perceptual size distortions that can be tested in virtual reality (VR) (e.g., the Moon Illusion and Sky-Dome Illusion, see Rudrauf et al., 2018-preprint). It also makes quantitative predictions about the role of projective imagination in enhancing behavioral flexibility, emotion-regulation, and resilience in relation to goal-directed behaviors. PCM agent simulations predict internal cognitive and affective states (which could be assessed with self-reports and performance metrics) as well as approach-avoidance behaviors, and more generally navigation in space (which could be linked to empirical data based on motion tracking) (cf., Figure 3’s simulations of a virtual pit challenge). Such predictions can be tested using VR, and we are currently implementing VR paradigms to do so. We note that the PCM also suggests a general framework for conceptualizing a range of psychopathological disorders and predicting their attendant mental states and behaviors (see Rudrauf and Debbané, 2018).

## The “Hard Problem”, Representationalism, and Phenomenal Selfhood

We turn finally to some philosophical issues that still preoccupy theorists of consciousness: the “Hard Problem”, representationalism, and the problems of subjective character or phenomenal selfhood. We discuss these in relation to the PCM.

### The “Hard Problem” and Computational Functionalism

We agree with Varela that the neurophenomenological approach (outlined in the section “Methodology” above), provides, in a certain sense, a “methodological remedy” for the “Hard Problem” of consciousness (Chalmers, 1996; Varela, 1996; Rudrauf et al., 2003). In our view, the method offers the best that “positive” science can do with regard to the problem of consciousness. In various guises, it has become more accepted in neuroscience (see, e.g., Oizumi et al., 2014), and it is perfectly in accordance with the quest for generative models (see, e.g., Petitot et al., 1999) such as the PCM; such models suggest ways to “axiomatize” the theory of consciousness, and thus go beyond mere correlations.

Neurophenomenology shares with classical Husserlian Phenomenology a bracketing of metaphysical positions, which are not decidable by phenomenological means. The metaphysics of consciousness cannot be decided by mere conceivability considerations either, since what is conceivable with respect to consciousness is constrained only by phenomenologically available, intuitive invariants. Its scope is correspondingly widened by what is phenomenologically *unavailable*; and it is fallacious to infer a property’s inapplicability to consciousness from its introspective unavailability (see, e.g., Williford, 2007; Elpidorou, 2016). This undermines the claim that phenomenology can determine the full metaphysical possibility space with respect to consciousness and explains how so many incompatible metaphysical positions could all seem equally conceivable. Thus framed, neurophenomenology is compatible, strictly speaking, with dualisms, panpsychisms, non-reductive physicalisms, and even with idealisms. It is thus also compatible with computational functionalism, a view that fits well with the PCM and is also independently motivated.

The PCM’s two main components can be fully described and implemented computationally or simulated (Rudrauf et al., 2017). In fact, on the assumption that the brain is an object in Euclidean-approximate 3-space, we *must* regard the PCM as a computational model, since its fourth dimension must then be a vector dimension computed algebraically with homogeneous coordinates. The PCM implies that the FoC relies on the straightforward computation of a four-dimensional structure that has no (non-computational) correlate in three-dimensional, ambient physical space. Does this mean that all simulated PCM agents are conscious entities?

In line with a computationalist version of *a posteriori* identity theory, we can consistently maintain that all instances of consciousness fit the PCM but that not all simulations of the PCM constitute a real consciousness.^17^ It is perfectly consistent and not particularly *ad hoc* to maintain that these simulations lack the necessary scale and organized complexity required for consciousness. If so, then it is not enough to create an artificial consciousness merely to implement the PCM in the truncated and highly abstract way required by practical simulations. But a system “running” the PCM that would more closely approximate the actual complexity, organization, and processing power of human or animal brains very well could, on our view, literally *be* an artificial consciousness.

This claim will no doubt bring to mind all the classic objections to computational functionalism (e.g., Block, 1978; Putnam, 1988; Searle, 1992; Buechner, 2008). But the phenomenological undecidability of the metaphysics of consciousness has an important implication here. In particular, it implies that our identity hypotheses about consciousness will involve the appearance of contingency in an ineliminable way.^18^ The force of many of the objections to computational functionalism turns precisely on this appearance of contingency. But once one accepts that the metaphysics of consciousness cannot be decided *a priori* or by appeal to phenomenology alone, one is no longer bothered by these apparently contingent elements that are knowable only *a posteriori*. This kernel of apparent contingency in the theory of consciousness can be rendered more palatable by proper reflection on structural features described in the computational model and the relations these features bear to phenomenological structures as well as to functional constraints. [One should also remember that even in pure mathematics there is the irremediable appearance of contingency (cf., Chaitin, 2003)]. One can thus think of this position as a combination of what is right about the functionalist tradition in philosophy of mind, artificial intelligence, and cognitive psychology with what is right about phenomenological realism.

Computationalist *a posteriori* identity theory allows that apparently contingent processing constraints may be essential to real consciousness. As long as the computational model captures the phenomenological invariants, meets the functional constraints, and survives the tribunal of prediction and experiment, we should happily accept that some apparently contingent and only empirically knowable constraints pertaining to the physical realization of consciousness are actually essential to it.^19^

The brain does indeed seem to be a kind of biological computer, among the other things it is (see Rudrauf, 2014). Barring the “wonder tissue” view of the brain (cf., Dennett, 2013) or some form of panpsychism, it is not at all implausible that it is (neuro)computational organization that principally differentiates consciousness from other brain processes. Moreover, it is not unreasonable to be an objective realist about such computation. In some way, the causal-informational organization of the brain allows it to *really* implement computations (i.e., realize the computation of certain functions objectively and in independence from the interpretations of observers). As long as one is realist enough about (approximate) mathematical structure in the world (and one should be, if one accepts physical laws as an apt summary of the world we live in), then accepting that brains (or computers, for that matter) *really* realize determinate computations ought not to be too large a pill to swallow.^20^

One may, of course, wonder further about the biophysics of brain computation, the brain’s “native coding language” so to speak, and, in particular, how the “real unity” of consciousness is achieved in this matrix. We have hypothesized elsewhere that the latter is achieved through a process that is equivalent to virtualization in computer science (see Rudrauf, 2014). And here we can reasonably expect that the general lesson of organismic biology is respected: preservation of form through the flux of matter. Form or structure requires material for its realization. In virtue of realizing that form, it gains certain causal powers (cf., Hofstadter, 2007), even the ability to reproduce the relevant form. We may not yet know in full detail how real, determinate computation is implemented in the brain, but there is little doubt that it is. Indeed, if one subscribes to the FE principle, some quantity is being optimized or computed through biophysical (neuronal) dynamics. And identifying consciousness with the realization of a certain computational structure poses, in principle, no *further* metaphysical conundrum in this regard. One might even reasonably suggest that conscious processing *just is* the relevant inferential processing (described here by the PCM) entailed by neuronal (and other) dynamics. The old notion (cf., Fodor, 1981) that functionalism (*modulo* phenomenological undecidability) provides the best solution to the Mind-Body Problem is still worth taking very seriously. It is certainly not *more* reasonable to take consciousness to be a fundamental, non-structural feature of reality. So far there have been no theoretical gains, measured by parsimony considerations in any case, derived from the recently passed vogue of neo-dualisms, nor from the current surprising (but also surely ephemeral) vogue of panpsychism and related views in analytical philosophy of mind (see, e.g., Brüntrup and Jaskolla, 2016).

We do not deny that the realization of consciousness may involve non-computational factors, even if, as we suggest, it involves some computational factors essentially. Again, we face the old problem of the iceberg here: consciousness is subtended by the unconscious, and our knowledge of the nature of the latter’s computations is limited. There are also issues surrounding continuity and discreteness: digital machines are thought of as discrete entities, but this a bad approximation of what happens in cells and in the physical world generally. We are embedded in the world, a reservoir of multiscalar complexity, which contains permanent, non-stationary motions and non-equilibrium states, including our own movements. We are in many aspects homogeneous with this world, which is not necessarily the case for computation as we currently tend to model it, even when many parameters are included. We are also confronted with difficult problems concerning the relations between energy and information. Moreover, as indicated above, a much wider kind of internal geometry underlies the Euclidean and projective geometries involved in action and consciousness; this internal geometry is made for action integration and thus takes into account energy and our physical interactions with the external world. We must simply accept that our understanding of the structure of computation, in relation to embedded geometries in the brain, is still very limited.

### The PCM and Representationalism

We want to forestall a worry that some representationalists might have about the way we have formulated the PCM.^21^ They might worry that we have committed a higher-order version of the “sense datum fallacy” (Prichard, 1938). According to that “fallacy”, one cannot infer from the appearance of, say, an orange-ish afterimage that *there exists* something that appears and *is* orange-ish. In fact, we are sympathetic with the Husserlian view that there exists something like sensory *hyle* so that, qualified the right sort of way, this inference is not fallacious (see Williford, 2013). But, indeed, to think of a unicorn does not imply that there exists a unicorn (of which one thinks); this is conceptual thought, however, not perception. Perception includes levels that are *beneath* the level of conceptual representation; and couched at some such “lower” level, the relevant *de re* quantification need not be illicit. Further, representationalists who want to identify phenomenal content with non-conceptual, intentional content have at the least to accept that there is some vehicular correlate of that non-conceptual content that one is indirectly but phenomenally aware of in having the content at all. Otherwise they cannot plausibly say what the difference is between two phenomenally conscious perception tokens that, by their hypothesis, must be aimed at non-existent property instances (cf., Hall, 2007; Thompson, 2008; Papineau, 2016). Thus, again, in a certain sense, we do not think the “sense datum fallacy” is actually a fallacy. Moreover, one can formulate a sort of representationalism, one that denies a strong form of its “transparency” thesis (cf., Kind, 2003), that converges with a more Husserlian model (see Williford, 2013, 2015).

However, one might yet object that we are making the following dubious inference: Consciousness represents space (and objects in it) in a projective manner; therefore, it *is* (or really approximates) a projective space. And here we would say roughly the same thing we say about sensory *hyle* or vehicles (Williford, 2013): at the very least the projective frame is a vehicular, structural property of consciousness. As such, in being aware of it, we are aware of properties *of* consciousness, not merely properties that consciousness represents.

Further, recall that essential properties of projective spaces are conspicuously missing from the Euclidean ambient space that we navigate via consciousness; the latter has no privileged origin, no points at infinity where parallels converge, etc. We can thus only make good sense of certain well known facts of perception (detailed above) on the assumption of a projective perceptual geometry. Representationalists, exhibiting some awareness of this issue, sometimes try to build these properties into first-order perceptual representations of physical objects [see, e.g., Tye, 2002 (in response to Peacocke, 1983, 1993); Van Cleve, 2002]. If this response is not a roundabout admission of defeat, it seems to border on the absurd, since it implies that we routinely misrepresent first-person experiential observer-object pairs as being entirely Euclidean *and* as having properties that *only* projective spaces have.^22^ It seems more elegant to make the projective structure into a general “vehicular” or “frame” property of consciousness *into which* various contents can be fitted, so to speak, and not to try to make these projective properties into covert properties of the objects represented.

We thus find our inference from projectively structured conscious *experience of the world* to a projective structure of *consciousness itself* not fallacious at all given the implausibility of the alternatives. Assuming that consciousness has this structure explains more—and more elegantly—than the competing representationalist hypotheses.

### The PCM, Subjective Character, and Phenomenal Selfhood

Subjectivity is given to us as a unified phenomenon. It is by reflection, analysis, and sometimes observation of clinical and psychopharmacological phenomena, that we learn to disentangle its many strands. Most salient for our purposes here are these elements: (i) the first-person point of view, (ii) pre-reflective self-consciousness, (iii) global self-consciousness, (iv) social self-awareness, (v) ipseity or “mineness” (foundational to the sense of body ownership and agency), (vi) the “transcendental ego”, (vii) the autobiographical self or “empirical ego.”^23^ We say a word about how the PCM relates and unifies many of these aspects of phenomenal selfhood.

The PCM begins with the situatedness of the conscious organism and the perspectival nature of experience. What we see from a given location at a given time; and we must multiply points of view on an object over time to extract a more absolute or objective conception of the object’s transphenomenal structure (which, again, is exactly what multi-view reconstruction algorithms are optimal for). This is encoded in the PCM in terms of the projective structure of the FoC. Further, the elusive “lived origin”, as we indicated above, must in fact arise from an inaccessible higher vectorial dimension. This elusiveness may seem to suggest a “viewer” or hidden “transcendental ego” and thus makes a certain “homuncularism” somewhat seductive. But this is just a structural feature of the conscious space.^24^ The origin cannot be experienced as a perceptual object. In that sense, it could never look at itself or catch its tail. In fact, there is no “thing” here that could “look at itself”, but rather a virtual pivotal point essential for the rendering of lived space, and necessary for establishing a direction of aim or “vectorization” of the FoC. It is not a detachable entity looking at an object. The real “transcendental ego”, so to speak, is not an ego at all. It is rather the unconscious computational machinery of the brain that is the generative mechanism of consciousness as a whole. The PCM thus gives a unified account of features (i) and (vi) above.

In addition to emphasizing the perspectival character of the perception of physical objects, the Phenomenologists were also wont to claim that consciousness is aware of itself in a pre-reflective and non-perspectival (or “absolute”) way (e.g., Zahavi, 1999; Sartre, 2004b). Even though one can take perspective on one’s own consciousness in reflection, memory and imagination, consciousness seems primitively self-aware without taking the kind of perspective on itself that it takes on objects of perception. It is not in need of a further consciousness taking a perspective on it in order to be aware of itself (our *Subjective Character* of the section “Phenomenological Invariants”). Moreover, it is aware of itself in some way as a unified whole (cf., our *Multimodal Synchronic* and *Temporal Integration* of the section “Phenomenological Invariants”).

The PCM suggests the following account of these features. First, the projective structure of the space of consciousness includes, formally, what we can call a “global reciprocity” property. This means that every point aimed at in the space from the origin, as it were, automatically “aims back” at the origin (without itself being an origin). This is not limited to just a single point attended to. It characterizes the entire phenomenal space no matter the degree of attention directed to a particular point. In that sense, the whole phenomenal space is experienced reciprocally and simultaneously from every point that appears in the space (even though at each instance one perspective dominates the framing). This is an internal property of projective geometry and not to be found in Euclidean geometry. It is manifest in a certain reversibility that is inherent in all conscious perception. In the case of seeing and hearing, the phenomenon is evident in what we might call the “reversibility of here and there.” To see or hear something as being “over there” is the same as the object’s appearing “(to me) over here.” In being conscious of the world, one is, so to speak, looking “back at” or “down upon” (the directional spatial metaphor does not matter since it is fully global) one’s consciousness of the world but from *within* consciousness itself.^25^ There is thus a kind of refle*x*ivity that is not representational in the usual sense, built in to the intrinsic structure of the conscious space. It is not a reduplication or new meta-representation of consciousness in the manner of usual higher-order theories or self-representationalism (see, e.g., Kriegel and Williford, 2006). It is perhaps better thought of as a form of self-acquaintance rather than self-representation (Williford, 2015).

There are two further important aspects of this global reciprocity. First, when coupled with the set of transformations that act on the projective space, global reciprocity grounds the moment-by-moment perspective taking capacities implicated in both working as well as deliberative perspectival imagination. The reciprocity of its structure and the relevant group of transformations together give to consciousness its horizon of possibilities—the sense of a set of points of view it *could* realize [something also well noted by Husserl (1982)].

The second point (cf., see the section “The Invariants and Projective Geometry in the PCM” above) is that this inherent sense of possible points of view is a major basis for intersubjectivity and social self-consciousness. If there are other possible points of view I can take, it is not too far a stretch for one to identify these with the actual points of view of others [an idea one can trace to Husserl and Edith Stein (see the discussion in Smith, 2016)]. In effect, once I have a sense of my actual, individual perspective vis-à-vis these other possible perspectives, I can, with the right cues (facial expressions, eyes), take one of these possible alternative points of view as being actually occupied by another—as if the other were “slotted into” one of my own possible points of view. If I did not experience the space surrounding me as being a field of possible other points of view, I could not experience it as being actually occupied by others with points of view on me. There is little doubt that a major function of consciousness and a factor in its evolution is that it allows one to imagine (whether in a working or deliberate way) oneself as seen from another point of view, friendly or unfriendly, as a ground for social cognition. These considerations allow us to unify the pre-reflective self-awareness built into consciousness as global reciprocity with social self-awareness or intersubjectivity. This relates aspects (ii) and (iv) above.^26^

Finally, there is the “ipseity” or “mineness” of consciousness [aspect (v)], which is equally important for grounding intersubjectivity, since one must be able to distinguish one’s own point of view from those of others and not merely think of others as extensions of oneself (see Zahavi, 2008 and many of the papers in Summa et al., 2018). This is sometimes characterized as the “*de se* constraint” on theories of self-consciousness (see Frank, 2007, 2016). A conscious being implicitly knows that it is *the very being it is*. This kind of fundamental acquaintance with oneself and one’s own individuality is not a matter of simply representing, via descriptions or some other mediated form of content, an object that one just happens to be. Nor can it be thought of as a matter of a conscious state bearing some sort of external or contingent relation to itself (cf., Frank, 2007, 2016). If it is relational at all, it must be a kind of “internal relation”, something grounded in the very nature of consciousness (Williford, 2015). We do not claim that the PCM, by itself, gives us a full solution this problem. Nonetheless, we can make a few remarks in this regard.

First, we do not have to accept haecceity views of primitive self-consciousness.^27^ If we make “mineness” into a fundamental property of consciousness as such and identify that with subjective character, then we have embraced, on this point at least, an essentially quasi-Leibnizian metaphysics of consciousness: something analogous to irreducible monads with individual essences. But subjective character is a generic property that all conscious beings share. Moreover, there is no reason to think that a conscious being’s complete set of *intrinsic* individuation conditions (*qua* consciousness) is something accessible, even though our individuality is indeed implicitly known and reflectively available. We are individuals in some intuitive sense, of course; but our sense of individuality can be parasitic on deeper aspects of the physical world that determine our objective spatio-temporal location and realization (see Williford, 2010, 2015). The sense of being a distinct individual, to which we do have reflective access, is, we think, ultimately derived from a deeper, inaccessible level—our “roots” in the physical world (cf., McGinn, 1993). Our lack of reflective access to these roots helps to give us an illusion of completeness and individuative self-sufficiency, since we tend to ignore or “fill in” all such blind spots.

We find ourselves as individuals thrown into the world and beings for whom “our being is in question”; this is a reflection of the fundamental individuating structures of the world and not itself one of those structures except in the trivial sense that we do indeed enjoy an awareness of our own individuality.^28^ If you like, consciousness knows itself immediately as an individual (or quasi-individual), but we cannot give a non-trivial, informative *ultimate* account of how and why it is that individual, which is as it should be, since *ultimate* individuation is either a matter of brute fact or of opaque logico-mathematical necessitation—neither of which makes much sense to us in this case, since neither could be derived from something more basic and intuitively obvious.

We might suggest then that among the roots of “essential indexicality” (Perry, 1979) or the *de se* constraint are these: the unrepresentability of a projective space (with its elusive origin) in a Euclidean space, which prevents us from experiencing ourselves as mere objects among others; the essential emptiness and conceptual impenetrability of our sense of individuality once it is stripped of all apparently contingent content (cf., Altobrando, 2018), which precludes reducing *de se* awareness to the *de re* or *de dicto* representation of an object as having properties (even haecceities); and our immediate, fundamental self-acquaintance (cf., Kripke, 2011), without which there could be no conceptually mediated *de se* awareness or representation in the first place [and, arguably, no *de re* or *de dicto* representation objects either (cf., Lewis, 1979; Chisholm, 1982)]. The first and last of these roots are directly implicated in the PCM; the second is, at bottom, a perfectly general matter having to do with the metaphysics and epistemology of individuation.

Does the PCM tell us anything about how we know ourselves in this immediate and non-representational way? The PCM entails that any realized conscious system should appear to itself in the reciprocal, non-conceptual way just described due to its intrinsic geometrical structure. It is just the very nature of this sort of structure; and there is no contradiction in holding a property to be both structural and intrinsic. Sufficiently interesting mathematical and computational systems can bear internal relations to themselves. In mathematical logic, to take a well known example, one finds purely abstract entities (certain formal axiomatic systems) that are well defined individuals and include, by their very structure, a sort of “window” into themselves (a well defined set of representable functions within the system that model the system itself and introduce a kind of self-reference). This is one of Douglas Hofstadter’s important insights vis-à-vis consciousness and computation (Hofstadter, 1979, 2007).^29^ As long as we are willing to imagine that a consciousness is a physically realized computational system that “models” itself just by its very structure, then we will have an internal, intrinsic relation (call it “self-modeling”) that holds between the system and itself of necessity and invariantly.^30^ If we are realist enough about the concrete, individualized implementation of computational structures in physical substrates (whatever their exact nature), then there is no barrier to being a realist about such a system. If we further identify (*a posteriori*) the realization of such a system with the realization of a phenomenal consciousness, then its “self-modeling” can, in principle, be identified with its self-manifestation (phenomenal self-acquaintance, minimal phenomenal selfhood), that is, the phenomenal manifestation of itself in its individuality. This point has an important application in the context of the PCM.

*Ex hypothesi*, the FoC as described in the PCM has a phenomenal manifestation. Thus this type of self-awareness structurally inherent in it has to be *phenomenal* as well, and not a matter of conscious inference or reflection or speculation or external relations one happens to bear to oneself: in being phenomenally aware of anything, it is *ipso facto* phenomenally aware of itself. All it will need then is a capacity for conceptual reification and quantification to begin thinking of itself as an individual with a history and a future, imbued with the functional fictions of personal identity (cf., Sartre, 2004b; Metzinger, 2011)—the “ideal” self, personal responsibility, *homo economicus*, the “neoliberal subject” etc.—with all the social structures built, for better *and* worse, upon them. These considerations integrate all of the aspects of self-awareness listed above (i–vii).

## Conclusion

We might divide contemporary neuroscientific and psychological theories of consciousness into three main types: Biofunctional (e.g., Varela, 1979; Edelman, 1989, 1992; Damasio, 1999, 2012), Cognitive-Neurofunctional (Baars, 1993, 1997; Dehaene, 2014), and Information Theoretic (Edelman and Tononi, 2000; Edelman, 2004; Tononi, 2004, 2005, 2008, 2011, 2012; Oizumi et al., 2014). As should be clear, the PCM incorporates the emphasis on self-awareness common to many approaches to consciousness^31^ as well as an emphasis on the biological functions of consciousness.

The PCM also incorporates the idea that global availability is one of the crucial functional features of consciousness, an idea common to Baars, Dehaene and others, if not the singly feature. This is built into our conception of the FoC, a virtual projective space in which various contents become accessible for further multimodal processing. The PCM gives a model of the computational substrate of global availability: it must involve optimizing multimodal information integration (via the FE principle), which populates consciousness with determinate content, and it must conform to a projective 3-space, which provides the overall organization of those contents, a precondition of availability.

We agree with Edelman and Tononi (2000) that information integration is also important to consciousness, but the treatment of information integration as formalized in the FE principle combined with the concept of FoC in the PCM suggests an important further articulation of the type of information integration specific to consciousness.

The PCM thus subsumes and unifies the main insights in all of these approaches to consciousness. Above all, what we add to preexisting theories is a geometry: the thesis that projective transformations and projective frames necessarily subtend the appearance and workings of consciousness.

## Author Contributions

DR: originator of the Projective Consciousness Model. KW, DR, DB, and KF: writing of sections “Introduction”, “Methodology”, “Phenomenological Invariants and Functional Features”, and “Conclusion”. KW: main writing of section “The ‘Hard Problem’, Representationalism, and Phenomenal Selfhood” (philosophical discussion). DB and KF: mathematical details. KW, DR, and DB: philosophical analysis and argumentation. DR and KF: neuroscience and psychology details. DR: design and implementation of the simulations discussed.

## Conflict of Interest Statement

The authors declare that the research was conducted in the absence of any commercial or financial relationships that could be construed as a potential conflict of interest.

## Footnotes

1 For readers interested in the model from a psychological and cognitive neuroscientific point of view and less interested in philosophical problems, the sections “Methodology” and “Phenomenological Invariants and Functional Features” can serve as a stand-alone introduction to the PCM. KW is primarily responsible for the more philosophical the section “The ‘Hard Problem’, Representationalism, and Phenomenal Selfhood”; anything dubious in it should not necessarily be held against the other co-authors.

2 We use the word “invariant” to denote the features of a phenomenon that remain the same throughout all transformations. The target phenomenon can itself be intrinsically dynamic (e.g., fluid flows, economic processes, consciousness itself) and yet exhibit invariances. In physics, these invariances are referred to as symmetries; these underlie nearly all of physics (e.g., gauge theories like General Relativity).

3 There is an extensive, multidisciplinary literature on each of these “invariants” spanning literally centuries. (Metzinger, 2003 is a fairly comprehensive place to begin; in relation to the PCM, see Williford et al., 2012 and Rudrauf et al., 2017.) Note that we *assume* that these features are invariants; together they constitute a set of working postulates. There is phenomenological and psychological evidence for each, but one can find, for each one, someone who has doubted its invariance. We do not think this matter can be resolved by thought experiment or other *a priori* methods, nor do we think it can be resolved by phenomenology or the interpretation of clinical data. All of these are relevant, but a full resolution will only come from a conceptually rigorous, empirically successful model. It could turn out that the PCM characterizes only normal, waking human and animal consciousness and not all possible consciousness as such. We have larger ambitions but the former would still be quite significant.

4 Note that this does not mean that we think that all forms of intentionality involve a so-called “real relation” between a real object and a subject (e.g., in thoughts about fictions), but we do hold that there is always a “real relational” layer in perceptual experience (see Williford, 2013) and that conscious experience (whatever the modality) does have a spatial structure organized around a point of view; this includes olfaction, audition, proprioception, mood states, nausea, and conscious “pure thought”, etc., even though the spatial structure may not be as obvious for these, at first glance, as it is for vision (see Williford et al., 2012; Chomanski, 2016; Kapitan, 2016). We cannot fully defend this claim here, but the basic idea is that all conscious “appearance-of” is “appearance-to” and that this latter is sufficient for phenomenal spatiality because that to which something appears is an “zero point” or “origin” in the sense of projective geometry. Thanks to Tad Zawidzki, Bartolomiej Chomanski, Peter Schulte, Jennifer Windt, and Dan Zahavi for discussions and debates on this point.

5 The elusive origin is not to be confused with, though it bears a certain relation to, the “cyclopean eye” of von Helmholtz and Hering (see, e.g., Turner, 1994 for history and Erkelens and Van Ee, 2002 *vs.* Khokhotva et al., 2005 for a recent debate). Note that the elusive origin subtends all modalities, not just vision. More importantly, the cyclopean eye’s origin, which can be precisely located through optometric measurements, must be inferred “from outside the subject” on the basis of data involving visuo-motor tasks and need not be assumed accessible introspectively. It is possible if not likely that the cyclopean eye’s optometric origin relates to the fact that the origin of the FoC often elusively feels to be behind the eyes in (especially visual) perception; but the projective model implies that it will not be precisely located in the space of consciousness as such. There is thus no contradiction between the optometric concept and the projective model. Thanks to Björn Merker for discussions on this point.

6 This is a bit like the Kantian view that experiential space is an “*a priori* intuition”; the difference is that for us the space in question is projective rather than Euclidean (cf., Friedman, 2012—thanks to Oliver Schliemann for this reference) or hyperbolic (as in Heelan, 1983—see Rudrauf et al., 2018-preprint for a comparison with Heelan’s model).

7 See, e.g., Hilbert and Cohn-Vossen, 1952, Coxeter, 2003, and Richter-Gebert, 2011.

8 The notion of a “projective frame” is a technical notion in the PCM that we do not have space to treat of in detail (see Rudrauf et al., 2017). Briefly, a 3D projective frame is defined by five points (such that no four are on the same projective plane): the center O (the “point of view”), three points at infinity (H on the horizon in front, L laterally, V vertically) and a fifth point, I, in the finite ambient affine 3-space, which behaves as a control point modulating the spatial scope of information integration (e.g., when one pays attention to an object “nearby” versus looking out on a wide-open vista from a high vantage point). See Rudrauf et al. (2017) and Rudrauf et al. (2018–preprint).

9 The old debate between Neo-Kantians and Phenomenologists about whether what we call “working perspectival imagination” is really properly considered a kind of imagination is, on our view, ultimately terminological (see, e.g., several papers in Summa et al., 2018). Note that this illustrates one specific way in which imagination and perception are integrated in our normal conscious experience. Deliberative or counterfactual imagination is integrated with perception in a *via negativa* manner: as Sartre (2004a) argued, it is (partly) on the foundation of current perception that one can intuitively know the difference between the real and the imaginary. See also Rudrauf et al., 2017 and Figure 4 below.

10 See the classic articulation of this problem in Ch. IV of Poincaré’s *Science and Hypothesis* in Poincaré, 2001.

11 We do not have space to treat the details of these accounts here (see references in text). Figures 1, 2 should be revisited after reading the section “Functional Features of Consciousness.”

12 The literature on each item is, again, multidisciplinary and massive. See Rudrauf et al. (2017) for references relevant to the PCM.

13 See Prinz (2012) for the dissenting view on this; his identification of attention with availability to working memory (pp. 90ff) suggests that his theory is based on what we regard as but one functional feature of consciousness (*Global Availability*) that is indeed central; perhaps some of the debate is just terminological. Thanks to Ali Yasar for discussion on this point.

14 We shall further demonstrate this in future reports (cf., Rudrauf and Debbané, 2018).

15 This formulation features an explicit quantity of information to be maximized expressed as an entropy and emphasizes the fact that the logarithm of prior probabilities behaves like a microscopic energy that becomes internal energy when summed on the posterior law.

16 This is why we do not frame the paper primarily in reference to known or suspected neurological structure-function relations (e.g., the dual stream hypothesis of Goodale et al., 1991 and Milner and Goodale, 2008; thanks to Björn Merker on this point). In Rudrauf et al., 2017, we do make some general hypotheses about neurological structure–function relationships and note that the PCM is certainly consistent with what is known about such relations.

17 By “computationalist *a posteriori* identity theory” we mean the view that (1) all instances of consciousness are token-identical to physically realized instances of possible phases in a generically identical computational process (program, software) C, and that (2) both C and the constraints on the realization of C (e.g., information processing speed, memory capacity) in a given substrate (hardware) must be determined *a posteriori*.

18 Indeed, this appearance of contingency will apply to *any* ontology of consciousness that is not vacuous (e.g., non-vacuous versions of substance dualism, idealism, or panpsychism). To think otherwise is to think that it is possible to know *a priori* that some substantive ontological theory is necessarily true of consciousness. But no mere mortal seems to be able to know this, even if a God could—which, evidently, does not help us in any case. (See, e.g., Williford, 2004, 2007, cf., Wilson, 2014.)

19 Something similar can be said vis-à-vis the problem of “qualia” (see Williford, 2013, 2015).

20 We accept a modest applied mathematical realism bearing well in mind the reasonable limitations and caveats that must inform such a position. For important and illuminating discussion, see Wilson (2006).

21 We should disclose here our general sympathy with the *internalist, indirect realist* orientation of many people working in the active inference, predictive coding, and Bayesian Brain paradigms (see, e.g., Anderson, 2017; Clark, 2017; Hohwy, 2017 for interesting recent discussion and Metzinger, 2003; Revonsuo, 2009 for more general arguments in favor of this orientation; see also Windt, 2018 for related food for thought).

22 It is not absurd for being an “error theory” but rather for the specific content of the error. See Hill, 2009, 156ff for a different, more plausible sort of representationalist response to this problem. Hill argues that we are normally just inattentionally blind to these projective properties of awareness.

23 The literature on these elements and their relationships is, of course, immense, multidisciplinary, and spans centuries (see, e.g., Zahavi, 1999, 2008; Metzinger, 2000, 2003; Frank, 2007, 2016; Kriegel, 2009; Williford et al., 2012; Zahavi and Kriegel, 2016; Billon, 2017; Guillot, 2017; Howell and Thompson, 2017).

24 The elusive origin of the perspective and the whole space for consciousness, are unified in projective geometry as follows: the projective group immanent in changes of frame are bijective one-to-one transformations of RP^3^, but the projectivities corresponding to perspectives, come from non-bijective 4D linear transformations. The latter have a kernel, the set of vectors that go to zero; on this kernel the transformations of lines have no sense. This kernel yields the elusive origin of the projectivity, but from one plane to another the map is well defined. Thus the unification of these two kinds of transformations is achieved by 4 × 4 matrices.

25 Note that this does not undermine the asymmetry between the point of view and objects or qualities intended or represented in the conscious space (cf., Merker, 1997); these latter are, so to speak, transient inhabitants of the affine compartment of the space. The global reciprocity property is invariant and subtends the whole space. Thanks to Björn Merker and Christian Nimtz for discussions on this point.

26 Though we cannot expand on this here, we note that the global involutive structure of a projective space may also contribute to the sense that consciousness is aware of itself as unified whole. It may also help explain the sudden sense of self-distance or scissiparity that occurs in hyper-reflective states, certain types of mystical experiences, depersonalization and derealization (see, again, Rudrauf et al., 2017). This would relate aspects (ii) and (iii) above.

27 Arguably Husserl held such a view, see, e.g., Zahavi, 1999. Chisholm (1982) held and then was convinced to abandon such a view by Dieter Henrich. Thanks to Manfred Frank for discussions on this point.

28 Thanks to Dan Zahavi and Miguel Sebastián for discussions on this point; they will likely disagree (for different reasons).

29 Cf., Varela, 1979, Metzinger, 2003, 2008, 2009, and Seth, 2014.

30 This satisfies Strawson, 2015 quest for a kind of relation (other than identity) that primitive self-consciousness could be.

31 An emphasis on self-awareness (variously understood) can be found in those neuroscientists, philosophers, psychologists, and cognitive scientists sympathetic with higher- and same-order representation and self-model theories of consciousness (see, e.g., Hofstadter, 1979, 2007; Lycan, 1996; Damasio, 1999; Carruthers, 2000, 2005; Flohr, 2000; Singer, 2000; Metzinger, 2003; Van Gulick, 2004; Rosenthal, 2005; Kriegel, 2009; Lau and Rosenthal, 2011; Gennaro, 2012; Sebastián, 2012; Weisberg, 2014).
